# Vitamins C and D Exhibit Similar Antidepressant Effects to Escitalopram Mediated by NOx and FKBPL in a Stress-Induced Mice Model

**DOI:** 10.3390/nu15122692

**Published:** 2023-06-09

**Authors:** Omar Gammoh, Aseel Ibrahim, Esam Qnais, Abdelrahim Alqudah, Sara Altaber, Alaa A. A. Aljabali, Murtaza M. Tambuwala

**Affiliations:** 1 Department of Clinical Pharmacy and Pharmacy Practice, Faculty of Pharmacy, Yarmouk University, Irbid 21163, Jordan; 2 Faculty of Sciences, Yarmouk University, Irbid 21163, Jordan; aseel.mane7@yahoo.com; 3 Department of Biology and Biotechnology, Faculty of Science, The Hashemite University, Zarqa 13133, Jordan; esamqn@hu.edu.jo (E.Q.); sara.taber12@gmail.com (S.A.); 4 Department of Clinical Pharmacy and Pharmacy Practice, Faculty of Pharmaceutical Sciences, The Hashemite University, Zarqa 13133, Jordan; abdelrahim@hu.edu.jo; 5 Department of Pharmaceutics and Pharmaceutical Technology, Faculty of Pharmacy, Yarmouk University, Irbid 21163, Jordan; alaaj@yu.edu.jo; 6 Lincoln Medical School, University of Lincoln, Brayford Pool Campus, Lincoln LN6 7TS, UK

**Keywords:** periostin, stress-induced mice model, mood disorders, vitamin C, vitamin D, alternative antidepressant drugs

## Abstract

The aim of this study was to investigate the potential antidepressant and anxiolytic effects of vitamin C and vitamin D in a stress-induced mouse model of depression, while also exploring the association between these effects and the levels of circulating NOx, periostin, and FKBPL. Our findings revealed that both vitamin C and vitamin D exhibited comparable antidepressant effects to escitalopram, a commonly used antidepressant, without demonstrating any anxiolytic effects. The antidepressant properties of vitamin C and vitamin D were linked to the normalization of Nox and FKBPL levels, while the levels of periostin showed no significant correlation. These results are consistent with previous research, indicating that the antidepressant effects of vitamin C and vitamin D may be attributed to their antioxidant and anti-inflammatory properties, as well as their modulation of neurotransmission and norepinephrine release. Additionally, our study uncovered elevated levels of periostin in stress-induced depression, which were only restored to normal levels by escitalopram, suggesting a potential role for periostin in mood disorders. Furthermore, FKBPL and NOx levels were increased in stress-induced depression and normalized by treatment with vitamin C, vitamin D, and escitalopram, indicating their involvement in the stress response and gene expression regulation. However, it is important to acknowledge certain limitations of our research, such as the use of a single depression induction model and limited dosing regimens. Future investigations should focus on examining these markers in specific brain regions, such as the hippocampus and prefrontal cortex, to gain a more comprehensive understanding of their potential implications for depression. Overall, our findings suggest that vitamin C, vitamin D, and escitalopram may possess antidepressant properties mediated by NOx and FKBPL levels, while emphasizing the potential significance of periostin in the context of depression.

## 1. Introduction

Psychological stress is related to physiological compensatory changes that include hormonal, endocrine, and other pathways [1,2]. More importantly, stress is related to other mood disorders, mainly depression and anxiety. According to the World Health Organization (WHO), major depressive disorder (MDD) is headed to be a major cause of disability in the world by 2030 [3]. Depression is not only highly comorbid with anxiety disorders and chronic diseases [4]. Despite the availability of different synthetic antidepressants, such as selective serotonin reuptake inhibitors (SSRIs), serotonin norepinephrine reuptake inhibitors (SNRIs), and tricyclic antidepressants (TCAs), which work by increasing the levels of monoamines at the synaptic cleft, their delayed onset and the inconsistency of antidepressant efficacy are major limitations [5].

Therefore, the need to evaluate the psychotropic roles of other molecules working through different mechanisms remains essential. The best candidate molecules should exhibit a well characterized safety profile, preferably nutrients. Mood disorders are tightly related to oxidative stress and inflammation. In this context, emerging evidence suggests a potential antidepressant role for vitamins C and D [6,7]. A randomized study reported the benefits of vitamin C supplementation in patients diagnosed with depression [8]. Similarly, vitamin D deficiency was seen in patients diagnosed with major depression: A randomized study reported the benefits of vitamin C supplementation in patients diagnosed with depression [8]. Similarly, vitamin D deficiency was seen in patients diagnosed with major depression [7]. Nitric oxide (NOx), which is produced from L-arginine by enzymatic conversion of the enzyme NOx synthase (NOS), is an important player in mood disorders [9]. NOx is increased in stress and depression [10,11], and NOS modulators have demonstrated antidepressant effects [7,12].

Nitric oxide (NOx), which is produced from L-arginine by enzymatic conversion of the enzyme NOx synthase (NOS), is an important player in mood disorders [9]. NOx is increased in stress and depression [10,11]. Moreover, NOS modulators demonstrated antidepressant effects [12].

Periostin is a protein that is usually associated with bone growth and repair. It may be a biomarker for anxiety and depression. Scientists have found that people with major depression or anxiety have more periostin in their blood than most people. Higher levels of periostin have been associated with more severe symptoms of depression and anxiety. The immune system’s interaction with periostin and its ability to promote inflammation may explain the role of periostin in these diseases. In mice, for instance, periostin has been linked to the development of anxiety-like behavior due to its activation of immune cells [13,14].

FK506-binding protein-like (FKBPL) is a multifunctional protein that belongs to the immunophilin family of proteins [15]. It was first identified in 1999 as a novel FK506-binding protein (FKBP) that exhibited homology with FKBP12, but with a different pattern of tissue expression. Since then, numerous studies have demonstrated that FKBPL plays a critical role in a variety of biological processes, including immune response [16], tumor growth [17], and angiogenesis [18]. Moreover, FKBPL is closely aligned with other members in a region where the tetra-trico-peptide repeat (TPR) domains are found. This is important for forming FKBP-associated molecular chaperone complexes, where it binds to heat shock protein-90 (Hsp90) via the TPR acceptor sites [19], which is important in regulating glucocorticoid receptors (GR), androgen receptors (AR), and oestrogen receptors [20,21]. Several studies showed that GR plays a crucial role in the regulation of stress responses [22,23], suggesting that FKBPL might have an important role in the pathogenesis of anxiety and depression.

Animal models are useful tools for investigating the neurobiology of psychological stress, as well as mental diseases, such as depression and anxiety. The acute restraint model, using immobilization as a stressor, is a reliable stress inducer [24,25]. The current study aimed at evaluating the potential antidepressant and anxiolytic roles of vitamin C and vitamin D with inflammatory markers, namely, NOx, periostin, and FKBPL, in a mouse model of stress.

The current investigation utilizes a unique methodology that explores the possible therapeutic benefits of vitamin C and vitamin D as antidepressants and anxiolytics in a mouse model subjected to stress. This research establishes the antidepressant effects of these vitamins, as well as their closeness to the widely used antidepressant medication escitalopram. Notably, the research fills a gap in the body of knowledge on this topic by concentrating on how the effects of these vitamins relate to the levels of NOx, periostin, and FKBPL in circulation. The results further clarify the crucial role played by periostin in mood disorders, as well as the contribution of FKBPL and NOx levels to the stress response and gene expression control. However, the limitations of the study, such as the use of only one depression induction model and limited dosing regimens, highlight the need for further study. Overall, by showing their similar efficacy to escitalopram and fewer side effects, this study sheds substantial light on the possible use of vitamins C and D as substitute antidepressant medications.

## 2. Materials and Methods

### 2.1. Animals

BALB/c mice (from the Animal House Facility of Yarmouk University, Irbid, Jordan) were used in the present study. The mice were aged 6–8 weeks and weighed 25 g. The BALB/c line was selected due to its suitability for achieving the study objective of enhancing citalopram efficacy. Mice were maintained in separate cages at a temperature of 25 °C with 50–60% humidity and continuous air ventilation. The research was conducted according to international ethical standards for the care and use of laboratory animals, and the study was approved by the Yarmouk University IRB committee and the Dean of Scientific Research Project Number (51/2022).

### 2.2. Study Design and Treatments

After a 2-day habituation period, mice were randomly assigned to one of six groups (*n* = 6–8 per group): naive (unstressed), control (stressed), escitalopram (10 mg/kg/day), ascorbic acid (10 mg/kg/day), vitamin D (1200 IU/kg/week), or diazepam (2 mg/kg/day) for 7 days. The dosages were chosen based on the relevant literature. On the 8th day, mice in all groups underwent acute immobilization stress for 8 h. Following the stressor exposure, the mice underwent behavioral testing and were then euthanized for analysis.

### 2.3. Acute Restraint Model

Stress was induced in mice by restraining them for 8 h without food or water, with only breathing allowed. This acute immobility stress test was performed with slight modifications, following the method described by Machawal and Kumar [25]. It is important to note that such procedures can cause significant discomfort and distress to animals, and therefore, the ethical guidelines for animal research must be strictly followed to minimize the potential harm to the animals.

### 2.4. Behavioral Paradigms

#### 2.4.1. Forced Swim Test

The forced swim test (FST) is a widely-used behavioral model for evaluating antidepressant-like activity in rodents [26]. Briefly, mice were individually placed in an open glass chamber (25 × 15 × 25 cm^3^) filled with fresh water to a height of 15 cm, which was maintained at a temperature of 26 ± 1 °C. The total duration of the test was 5 min. Floating time (FT) was defined as the period in which mice remained completely immobile in the water. This parameter was used to assess the antidepressant-like activity of the test compounds.

#### 2.4.2. Tail Suspension Test

The tail suspension test is a widely used method for evaluating depressive-like behavior in rodents [27]. To conduct this test, mice were suspended individually 50 cm above the floor using adhesive tape placed approximately 1 cm from the tip of their tail. The immobility time during the last 5 min of the 6-min test period was measured. This method provides a reliable measure of the animal’s behavioral response to stress and can help assess the efficacy of potential antidepressant treatments.

### 2.5. Elevated Plus Maze

Elevated Plus Maze: The Elevated Plus Maze (EPM) test is a widely used model for screening anxiolytic compounds in rodents [24]. In this study, we performed the EPM test with slight modifications, as previously described. Briefly, the maze was elevated 25 cm above the floor and consisted of two closed arms (30 × 5 × 10 cm) and two open arms (30 × 5 cm). Mice were placed at the center of the maze, facing the closed arm, and allowed to move freely for 10 min. The frequency of Open Arm Entries (OAE) and the duration of time spent during the Open Arms Test (OAT) were recorded by an experienced technician.

### 2.6. Open Field Test

An OFT was performed to assess the locomotion, anxiety, and sedation in the mice [28]. Briefly, the mice were placed in a central square and allowed to move freely for 5 min. The field was in a test room and lit by indirect lighting. The procedure was performed in an empty room to minimize noise and distractions. The open field maze was cleaned between each mouse using 70% ethyl alcohol. The locomotion activity (represented by the Ambulation Frequency (AF)) and the sedation (represented by the rearing frequency) were recorded.

### 2.7. Western Blots

Western blot analysis was performed according to standard protocols with some modifications [29]. Mirzaii-Dizgah et al. (2020) with some modifications. Total protein was quantified using a bicinchoninic acid assay kit (Bioquochem, Parque Tecnológico de Asturias Edificio Ceei, Spain), and equal amounts of protein were separated by sodium dodecyl sulfate-polyacrylamide gel electrophoresis (SDS-PAGE). The protein was then transferred to a nitrocellulose membrane (Thermo Fisher Scientific, Darmstadt, Germany). The membrane was blocked with 3% bovine serum albumin (BSA) for 1 h at room temperature before incubating overnight with primary antibodies against periostin and FKBPL (Abcam, Cambridge, UK). After three washes with washing buffer (Tween-20 or Tris-buffered saline), the membrane was incubated with the appropriate secondary antibody (Mybiosource, San Diego, CA, USA) for 1 h at room temperature. Following three additional washes, the membrane was incubated with an ECL substrate (ThermoScientific, Darmstadt, Germany) for one minute and then imaged using a chemiLITE Chemiluminescence Imaging System (Cleaver Scientific, Rugby, UK). Equal gel loading was confirmed using -actin as a housekeeping gene (Mybiosource, San Diego, CA, USA). The intensity of the bands was measured using ImageJ software version 1.8.0_112.

### 2.8. Nitric Oxide Assay

Blood samples were collected from the mice, and serum was prepared following standard protocols. The production of nitric oxide (NO) was measured by quantifying the accumulation of nitrate using a colorimetric assay with Griess reagent [30]. Serum nitrate was quantified using a Nitric Oxide Assay Kit (Sunlong, Hangzhou, China) following the manufacturer’s instructions.

### 2.9. Statistical Analysis

The data obtained from the behavioral tests, NOx, and protein markers were analyzed using a one-way ANOVA followed by Tukey’s post hoc test. The significance level was set at *p* < 0.05. The results are presented as the mean ± standard error of the mean (SEM).

## 3. Results

### 3.1. Forced Swim Test

**Figure 1:**
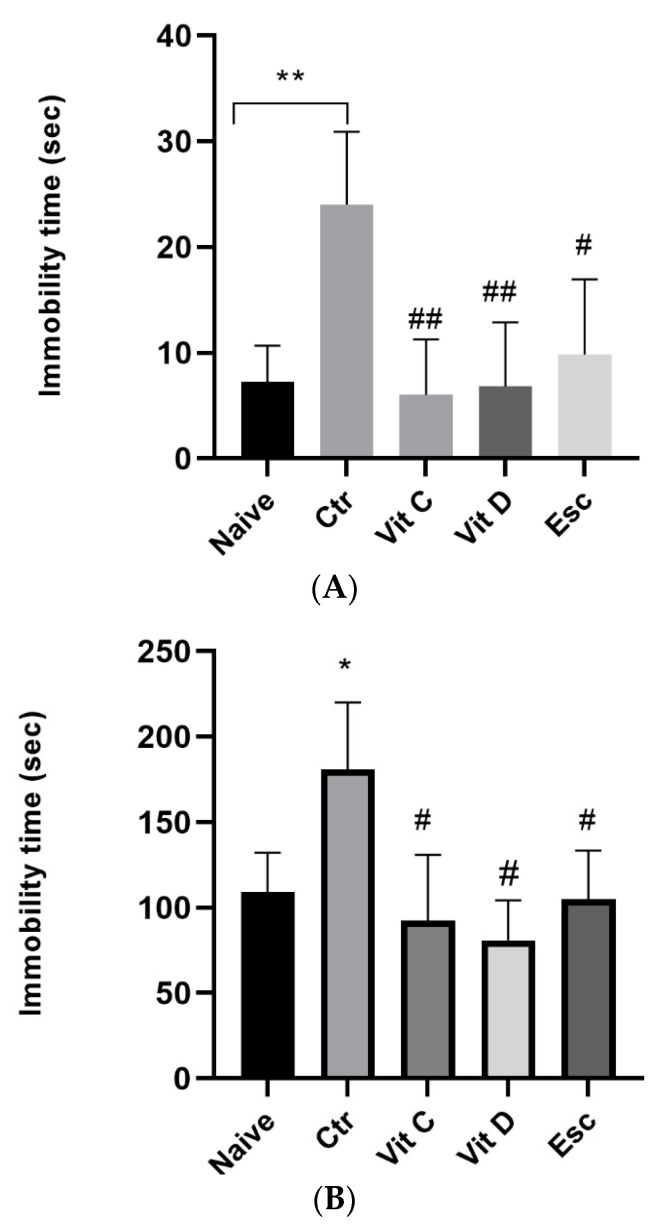
(**A**) Shows the floating time among different groups. The data were analyzed by one-way analysis of variance (ANOVA), followed by Tukey’s post hoc test. The results are presented as the mean ± standard error of the mean (SEM). The statistical analysis showed a significant difference among the groups (F(4, 21) = 5.48; *p* = 0.003). ** *p* < 0.007 compared to the Naive group, # *p* < 0.05 compared to the Control (Ctr) group, and ## *p* < 0.007 compared to the Ctr group. The abbreviations used are Ctr for control, Vit C for vitamin C, Vit D for vitamin D, and Esc for escitalopram. (**B**) Immobility time for the tail suspension test comparison among different groups in the forced swim test. Values are presented as the mean ± SEM (ANOVA followed by Tukey’s test). F(4, 22) = 4.48; *p* = 0.006, * *p* < 0.05 vs. Naïve, # *p* < 0.05 versus (Ctr). Ctr: control; Vit C: vitamin C; Vit D: vitamin D; Esc: Escitalopram; SEM: standard error of the mean.

Vitamin C and vitamin D were tested for their potential antidepressant effects using the forced swim test (FST). FST is a commonly used behavioral test in rodents to screen for antidepressant-like action. The measurement of the floating time (FT) was conducted, which refers to the duration during which the mice exhibited no movement while immersed in water. The findings of the Forced Swim Test (FST) revealed that the acute immobilization caused a noteworthy elevation in the Floating Time (FT) in comparison to the mice that were not subjected to any prior treatment (*p* < 0.05). The results indicate that the mice exhibited elevated levels of behavior consistent with depression after the administration of the forced swim test. Nonetheless, administration of vitamin C, vitamin D, and escitalopram demonstrated a noteworthy reduction in the FT (*p* < 0.05) in comparison to the control. The observation implies that the provision of vitamin C and vitamin D could potentially elicit antidepressant-like outcomes akin to those of escitalopram (Figure 1).

### 3.2. Tail Suspension Test

Vitamin C and vitamin D were tested for their potential depressive effects using the Tail Suspension Test (TST). Mice were tested by suspending them horizontally from the adhesive tape around 1 cm from the tail and 50 cm from the floor. The findings of the TST revealed that the acute immobilization caused a noteworthy elevation (*p* < 0.05) in the immobility time (IT) compared to the naïve. Additionally, vitamin C and vitamin D exhibited antidepressant effects similar to those of escitalopram (Figure 1).

### 3.3. Elevated Plus Maze

**Figure 2:**
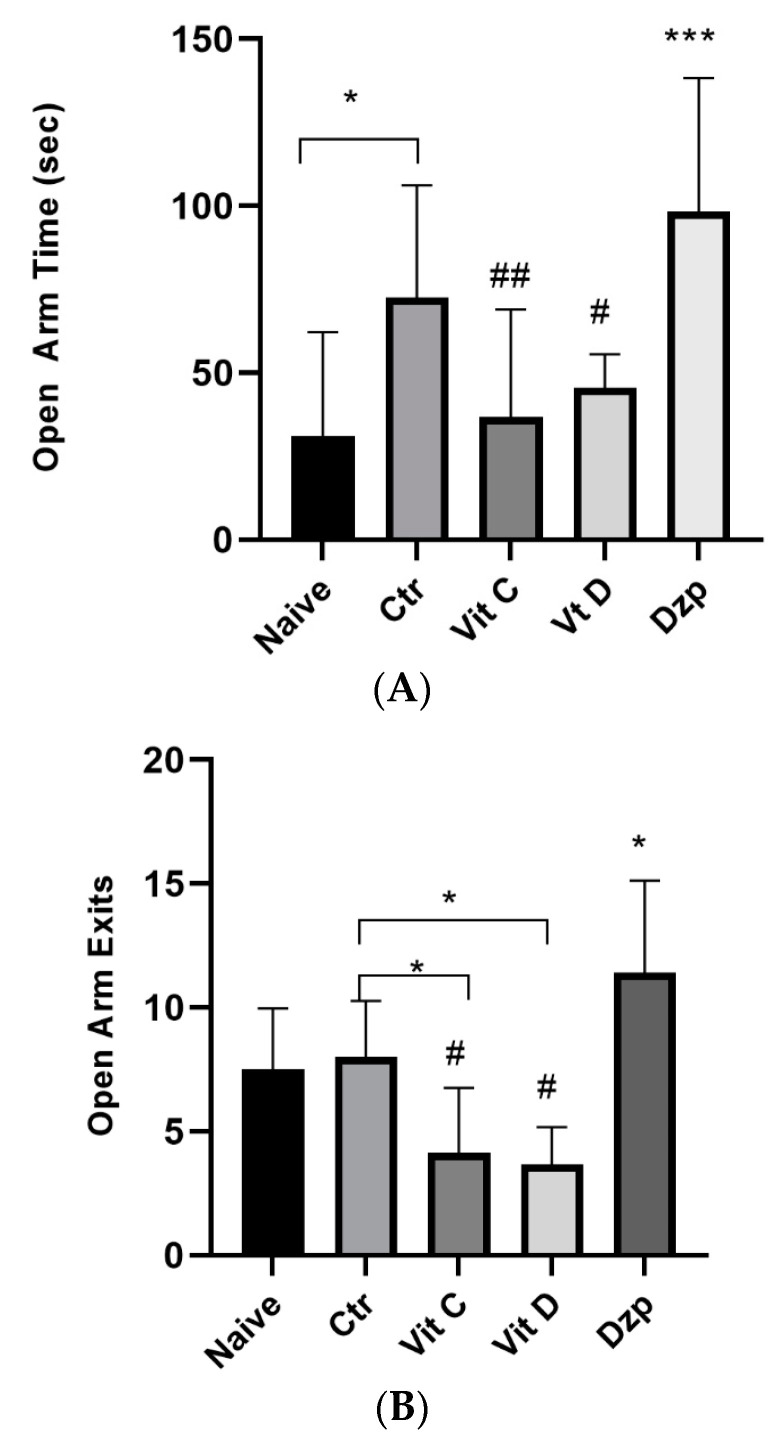
(**A**) Open arm time of the elevated plus maze test among the different treatment groups. Values are expressed as the mean ± SEM (ANOVA followed by Tukey’s test). F (4, 39) = 7.37, *p* = 0.0002. * *p* < 0.05 versus Naïve, *** *p* < 0.001 versus Naïve, # *p* < 0.05 versus Dzp and ## *p* < 0.001 versus Dzp. Ctr: control; Vit C: Vitamin C; Vit D: Vitamin D; Dzp: Diazepam; SEM: standard error of the mean. (**B**) Open arm exits among the different groups. ANOVA followed by Tukey’s post hoc analysis. Values are expressed as mean ± SEM (ANOVA followed by Tukey’s test). F (4, 41) = 11.27; *p* < 0.0001, * *p* < 0.05 versus control, # *p* < 0.0001 versus diazepam. Control (Ctr), vitamin C (Vit C), vitamin D (Vit D), and diazepam (Dzp) were used as treatments. SEM indicates standard error of the mean.

The control mice showed a significantly higher OAT (*p* < 0.05) compared to the control. The vitamin C and vitamin D groups showed a significantly lower OAT (*p* < 0.05) compared to the control mice. Moreover, diazepam-treated mice demonstrated a significantly higher (*p* < 0.05) OAT compared to the naive group. In regard to the OAE, vitamin C and vitamin D-treated mice demonstrated a significantly lower (*p* < 0.05) OAE compared to the control group; conversely, diazepam-treated mice showed a significantly higher (*p* < 0.05) OAE compared to the control group, as shown in Figure 2.

### 3.4. Open Field Test

**Figure 3:**
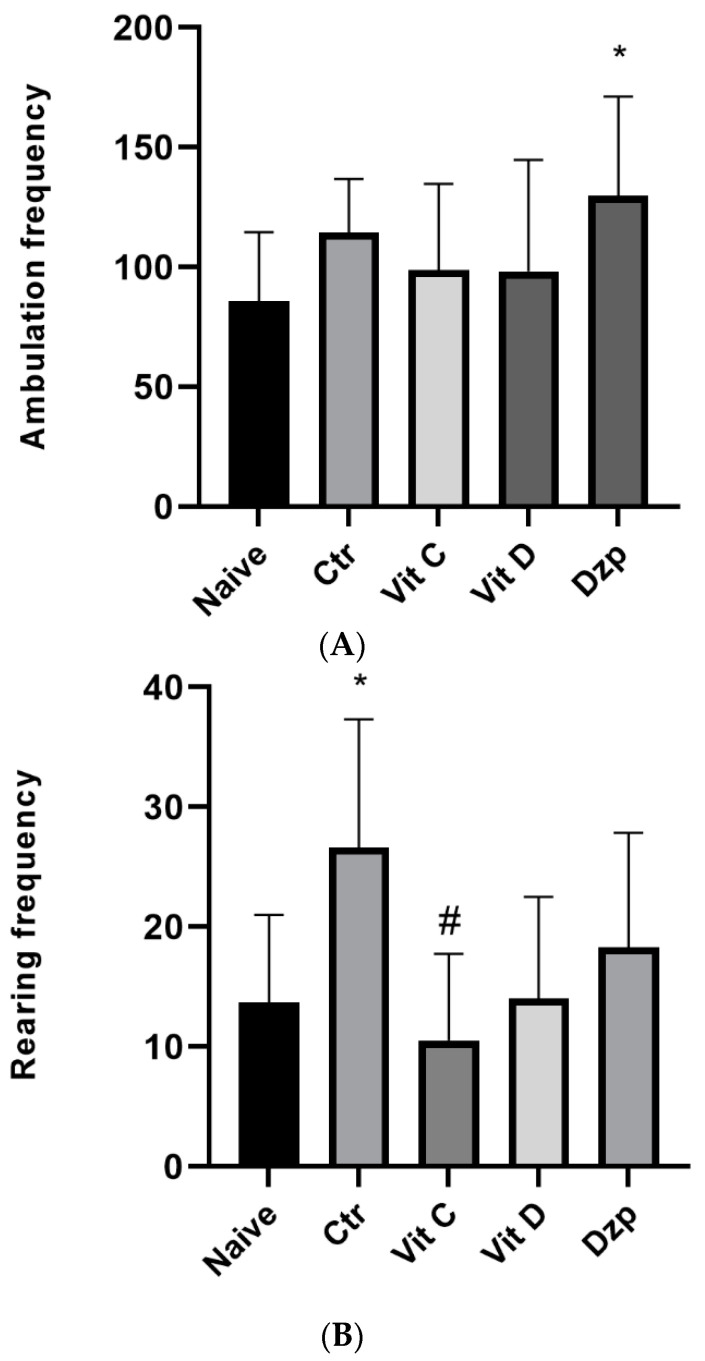
(**A**) Ambulation frequency among different treatment groups. Values represent the mean ± SEM, and statistical analysis was performed using ANOVA followed by Tukey’s post hoc test. F(4, 42) = 2.48; *p* = 0.04. * *p* < 0.05 versus Naive. Ctr: control; Vit C: Vitamin C; Vit D: Vitamin D; Dzp: Diazepam; SEM, standard error of the mean. (**B**) Rearing frequency among the different groups. ANOVA followed by Tukey’s post hoc analysis was performed. Values are expressed as the mean ± SEM. F (4, 33) = 4.33; *p* = 0.006. * *p* < 0.05 versus Naive, # *p* < 0.05 versus Ctr. Ctr: control; Vit C: Vitamin C; Vit D: Vitamin D; Dzp: Diazepam. SEM denotes standard error of the mean.

The mice’s mobility, anxiety, and level of sedation were all measured using the Open Field Test (OFT). The mice in this experiment were given 5 min of free reign within a square enclosure. Ambulation Frequency (AF) and Rearing Frequency (RF) were calculated to measure the amount of movement and rest, respectively. The OFT showed that, compared to the control group, the AF and rearing frequency were not substantially different for those who were given vitamin C and vitamin D. Figure 3 represents the major finding of this section.

### 3.5. Nitric Oxide (NOx)

**Figure 4:**
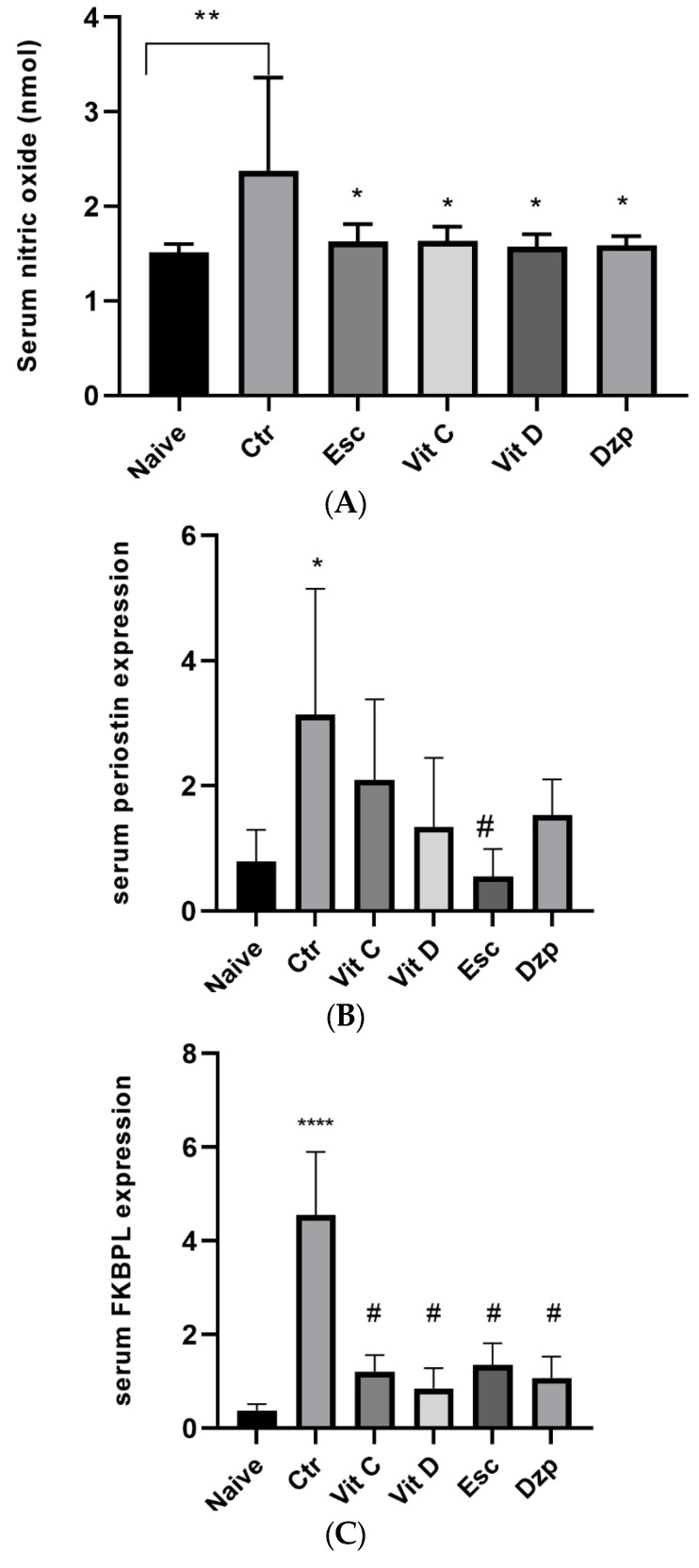
(**A**) Serum nitric oxide expression among the different groups. The values are presented as the mean ± SEM and were analyzed by ANOVA followed by Tukey’s post hoc test. F(5, 35) = 3.98; * *p* < 0.05 versus Naive, ** *p* = 0.005 versus Ctr. Ctr: control; Vit C: Vitamin C; Vit D: Vitamin D; Dzp: Diazepam; SEM: standard error of the mean. (**B**) Serum periostin expression among the different groups. ANOVA followed by Tukey’s post hoc analysis. Values are expressed as the mean ± SEM. F(5, 20) = 3.10; * *p* < 0.05 versus Naive, # *p* < 0.05 versus Ctr. Ctr: control; Vit C: vitamin C; Vit D: vitamin D; Esc: escitalopram; Dzp: diazepam. SEM indicates standard error of the mean. (**C**) Serum FKBPL expression in different groups. ANOVA followed by Tukey’s post hoc analysis. Values are expressed as mean ± SEM (ANOVA followed by Tukey’s test). F(5, 19) = 21.38, *p* < 0.0001. **** *p* < 0.0001 versus Naive, # *p* < 0.0001 versus Ctr. Ctr: Control; Vit C: Vitamin C; Vit D: Vitamin D; Esc: Escitalopram; Dzp: Diazepam. SEM: Standard Error of the Mean.

Nitrate buildup, an indirect measure of NO generation, was measured with a Griess reagent colorimetric test. The concentration of nitrate in the serum was significantly higher (*p* < 0.05) in the stressed group compared to the control, and nitrate levels were normalized in all treated groups similar to the naive group (*p* > 0.05). The data are shown in Figure 4.

### 3.6. Periostin

The control group (stressed untreated) demonstrated a significant increase (*p* < 0.05) in periostin expression compared to the naive group. Additionally, the escitalopram-treated group showed a significant reduction (*p* < 0.05) in periostin levels compared to the control group (Figure 4).

### 3.7. FKBPL

The control group (stressed untreated) demonstrated a highly significant increase (*p* < 0.0001) in FKBPL expression compared to the naive group. Additionally, all the treated groups showed a highly significant reduction (*p* < 0.0001) in FKBPL levels compared to the control group (Figure 4).

## 4. Discussion

We used a model of stress-induced depression and anxiety in mice to assess the potential antidepressant and anxiolytic effects of vitamins C and D. The correlation between the observed behavior and blood levels of NOx, periostin, and FKBPL was also examined. Similar to escitalopram, vitamin C and vitamin D were found to have antidepressant effects, and these benefits were linked to improvements in circulating levels of NOx and FKBLP, but not periostin. Significantly, neither vitamin C nor vitamin D showed any anti-anxiety effects.

The results presented here are in line with those found in the literature to date. In the tail suspension test and the forced swim test, for instance, vitamin C was found to have antidepressant effects, similar to those of antidepressants (fluoxetine and imipramine) [27]. Several mechanisms can explain this effect, such as modulating the neurotransmission of monoamines by modulating the binding of neurotransmitters to receptors [31] or preventing the release and reuptake of neurotransmitters [32]. Additionally, vitamin C was found to enhance the release of norepinephrine [33], a mechanism that is partly similar to that of antidepressants. Furthermore, since depression is closely related to oxidative stress and inflammation, vitamin C’s potent antioxidant and anti-inflammatory properties can explain its antidepressant properties [34].

Similarly, vitamin D has been linked to depression, and low vitamin D levels have been linked to depressive symptoms [7]. In line with our findings, previous studies showed that acute [35], and chronic administration of vitamin D in animals [36] resulted in a reduction in the immobility time in the forced swim test. Moreover, vitamin D deficiency in mice resulted in depressive symptoms [37]. Vitamin D’s antidepressant effect may be due to its antioxidant qualities, neuroimmunomodulation, or neuroplasticity, however, the evidence for this is still preliminary [38,39].

In the current study, stress-induced depression was found to increase periostin, and only escitalopram normalized it. This discovery establishes a link between antidepressants and periostin for the first time. Despite the lack of research, there is evidence that periostin plays a role in mood disorders through its involvement in axon regeneration and neuronal plasticity in the cerebral cortex [40].

Alternatively, vitamin C, vitamin D, and escitalopram were able to normalize the elevated FKBPL and NOx levels seen in stress-induced depression. Similar to other family members, FKBPL shares a region containing the TPR domains, with which it is highly aligned. This is critical for maintaining the steroid receptor in a conformation that allows fast ligand binding and transactivation of steroid-responsive genes, and it occurs as part of FKBP-associated molecular chaperone complexes, in which it binds to Hsp90 via the TPR acceptor sites [41]. Glucocorticoids, such as cortisol, are stress hormones that interact with GRs in the brain, which play a crucial role in the regulation of the stress response, and their dysfunction has been implicated in the pathogenesis of stress-related disorders, such as anxiety and depression [42,43]. In its unbound form, GR stays in the cytoplasm as part of a large multiprotein complex, including chaperones (Hsp90 and Hsp70) and immunophilins, including FKBPL [44]. Upon binding to cortisol, conformational changes occur, leading to the dissociation of GR from the multiprotein complex, before the activated ligand-bound GR complex translocates to the nucleus, where it regulates gene expression [45]. Studies have found that overexpression of the TPR peptide prevented effective nuclear accumulation of the GR [46]. Our results in this study showed an increase in FKBPL levels in response to stress, and this increase was abolished with treatment with vitamin C, D, and escitalopram. While NOx is extensively studied in stress and depression, this is, to our knowledge, the first report to show a role for FKBPL in such disorders, suggesting that FKBPL upregulation is associated with stress and depression disorders.

Similar antidepressant effects to escitalopram were seen with vitamin C and vitamin D, with normalization of circulating NOx and FKBPL, but not periostin. The study also discovered that vitamin C and vitamin D showed no signs of having any anxiolytic effects. Under stress-induced depression, periostin levels rose, and only escitalopram brought them back down to normal. Vitamin C, vitamin D, and escitalopram were able to correct stress-induced depression’s elevated levels of FKBPL and NOx. The overexpression of FKBPL was connected to mental health issues, such as anxiety and depression. The results of the investigation were consistent with what had already been discovered. The antidepressant effects of vitamins C and D can be explained by their powerful antioxidant and anti-inflammatory activities. Evidence of vitamin D’s role in depression is still developing; however, vitamin D deprivation in mice led to depressive symptoms. Mood problems may be related to the role of periostin in axon regeneration and neuronal plasticity in the cerebral cortex. The FKBPL gene is involved in the regulation of the stress response, and its malfunction has been linked to the etiology of anxiety and depression.

### Study Limitations

Although this study has a number of strengths, including its thorough methodology and precise choice of biomarkers, there is room for additional research to better understand the intricate factors that play into the development of depressive disorders. The lipopolysaccharide model, which includes both depression and inflammation, is one such model that could be investigated. In addition, the links between the biomarkers and depression may be better understood by investigating alternative combinations of therapies via varied dosage regimes.

Future studies should examine NOx, periostin, and FKBPL in the hippocampus and prefrontal cortex, two critical brain regions involved in depression, to provide a full picture of how these biomarkers are related to depressive symptoms. Studying these areas may lead to the identification of new intervention targets and a better understanding of the underlying mechanisms that contribute to depression.

Although the current study sheds light on how NOx and FKBPL levels may mediate the antidepressant effects of vitamin C, vitamin D, and escitalopram, more investigation is needed. Additional brain regions should be analyzed, and different models of depression induction and dosing strategies should be tested. New and improved treatments for depression may be possible because of this study, which will hopefully increase our knowledge of the complicated mechanisms behind this condition.

## 5. Conclusions

In summary, the antidepressant effects of vitamin C, vitamin D, and escitalopram are facilitated by the modulation of circulating NOx and FKBPL levels. The study confirms the role of periostin as an important mediator in depression and its normalization by escitalopram. The findings are consistent with previous research that has established the antidepressant potential of vitamin C and vitamin D. The mechanisms underlying the antidepressant properties of these vitamins are believed to be due to their antioxidant and anti-inflammatory properties, as well as their modulation of neurotransmission and release of norepinephrine. Specifically, the antidepressant effects of vitamin C and vitamin D were linked to the normalization of NOx and FKBPL levels, rather than periostin levels. Furthermore, periostin was observed to increase in stress-induced depression, and only escitalopram was effective in normalizing its levels. This highlights the potential role of periostin in mood disorders. On the other hand, the increased levels of FKBPL and NOx in stress-induced depression were normalized by the administration of vitamin C, vitamin D, and escitalopram. This indicates their involvement in the stress response and the regulation of gene expression. The study suggests that vitamin C, vitamin D, and escitalopram may offer alternatives to conventional antidepressant drugs, as they have comparable antidepressant effects with fewer side effects. Nevertheless, additional research is needed to validate these findings and explore the optimal dosages and administration routes of vitamin C and vitamin D in treating depression. In conclusion, the study establishes the potential antidepressant effects of vitamin C and vitamin D mediated by NOx and FKBPL levels, as well as the crucial role of periostin in depression. The study highlights the potential for these vitamins to offer alternative therapies for depression treatment, but further investigation is necessary to confirm these findings.

## Data Availability

Data is available with the corresponding author and can be supplied upon request.
